# Mapping knowledge structure and global status of sarcopenia in geriatric hip fractures: A bibliometric and visualized study

**DOI:** 10.3389/fsurg.2022.1019985

**Published:** 2022-10-05

**Authors:** Zhibang Zhao, Wenliang Fan, Qingbo Chu

**Affiliations:** Emergency Trauma Center, Nanyang Second People’s Hospital, Nanyang, China

**Keywords:** sarcopenia, elderly, hip fracture, bibliometric study, visualized study

## Abstract

**Background:**

Sarcopenia in geriatric hip fractures is attracting increased attention in recent years. This study aimed to explore the bibliometric characteristics and current status of sarcopenia research in hip fractures of older patients.

**Methods:**

Publications related to sarcopenia in geriatric hip fracture published between January 2000 and July 2022 were extracted from the Science Citation Index Expanded, and bibliometric and visualized studies were performed by VOSviewer, Citespace, and R.

**Results:**

The 1,223 articles used in our study were written by 6,326 authors from 1,879 organizations in 60 countries, published in 388 journals, and cited 37,198 references from 5,422 journals. The United States contributed the most publications (288 publications). The journal with the largest number of papers was Osteoporosis International (62 publications), and the Journals of Gerontology Series A - Biological Sciences and Medical Sciences had been more cited than any other journals in this field (3,302 citations). The University of Melbourne published the biggest number of papers (72 publications) focusing on sarcopenia in geriatric hip fractures while the California Pacific Medical Center Research Institute had the largest citations (4,239 citations). Cawthon PM was the most productive and influential author in the field. keywords were classified into 6 clusters: Cluster 1 (sarcopenia in aging), Cluster 2 (osteoporosis), Cluster 3 (bone density), Cluster 4 (body composition), Cluster 5 (physical performance), and Cluster 6 (risk factor).

**Conclusion:**

Geriatric hip fracture is one of the most significant health issues in the aging society. In the past 20 years, an increasing number of studies were performed to explore the relationship between sarcopenia and hip fracture in older adults. The United States showed the strongest influence in this field, including publication numbers, citations, institutions, funding agencies, and authorship. Sarcopenia in aging, osteoporosis, bone density, body composition, physical performance, and risk factors may become the future hotspots in this field.

## Introduction

Geriatric hip fracture as one of the most common and deadly types of fracture has an increasing incidence in recent years and has become a severe global health problem due to the aggravation of the aging society (1). Geriatric hip fracture is characterized by high mortality and disability rate: only 43% to 51% in men and 61% to 67% in women may survive 8 years after a hip fracture (2). The direct causes of hip fracture in older adults are usually low-energy injuries, such as falls and slipping (3). However, the most fundamental and essential pathway of geriatric hip fractures is the loss of bone mass density (BMD) in the aging body (4). Bone metabolism is a precise and complex procedure mainly regulated by two opposite procedures: bone regeneration and bone absorption (5). Many factors and pathways may associate with bone metabolism and result in the progress of osteoporosis (6). At the same time, bone metabolism is also related to the prognosis of geriatric hip fractures. Therefore, more and more researchers are focusing on the potential risk factors for bone regeneration and osteoporosis nowadays, and it is significant to identify the frontiers in hip fracture.

Sarcopenia is an aging-related disease characterized by loss of muscle mass, muscle strength, and physical function (7). The concept of sarcopenia was first put forward in 1989 by Irwin Rosenberg (8) and attracted lots of attention from researchers and doctors in the field of geriatrics. Nowadays, the causes and pathogenesis of sarcopenia are still not fully understood, and it is believed that some factors may contribute to sarcopenia including a long stay in bed, malnutrition, and denervation of muscle (9). Sarcopenia has been identified as an independent risk factor for many factors and is significantly associated with the mortality of older people, especially individuals with musculoskeletal disorders (10, 11). In recent years, more and more studies suggested that sarcopenia played a significant role in not only the incidence of osteoporosis and hip fracture but also the prognosis after hip surgeries (12, 13). Moreover, it is also reported that older patients who underwent surgeries for hip fractures may have an increased risk of sarcopenia, which may be caused by poor mobility and a long stay in bed after surgery (14). Despite much new knowledge in this field having been explored, there is still no consensus on the relationship between sarcopenia and hip fracture, and the underlying pathomechanism between them.

A great number of studies about the relationship between sarcopenia and hip fractures have been published in recent years (15, 16). However, it is difficult for researchers to keep up with the newest understandings and findings in this field when they are facing an increasing number of studies. Bibliometric analysis as a new and developing subdiscipline of library and information science can provide a quantitative and qualitative way to assess the research trends and identify the frontiers in a field by capturing the bibliometric characteristics (17, 18). The distribution structure, quantitative relations, change rules, and quantitative management of publications could be revealed and summarized by methods of mathematics and statistics in bibliometric studies (19). However, few studies explored and summarized the characteristics and research topics of studies about sarcopenia in geriatric hip fracture. In this study, we aimed to analyze the literature in this field from 2000 to 2022 to understand the current trends and topics of sarcopenia in geriatric hip fracture by using bibliometric methods, and to provide a comprehensive overview for researchers to explore this field and perform further studies.

## Material and methods

### Data source and search strategies

Web of Science (WoS, Clarivate Analytics, Philadelphia, PA, United States) as one of the most comprehensive academic database platforms was used to obtain the data and characteristics of publications (20). In our study, all of the documents were extracted from the Science Citation Index Expanded (SCI-E) database of the Wos Core Collection (WoSCC) on July 21, 2022. The publication time of the literature was limited between January 1, 2000, and July 1, 2022.

**Figure 1:**
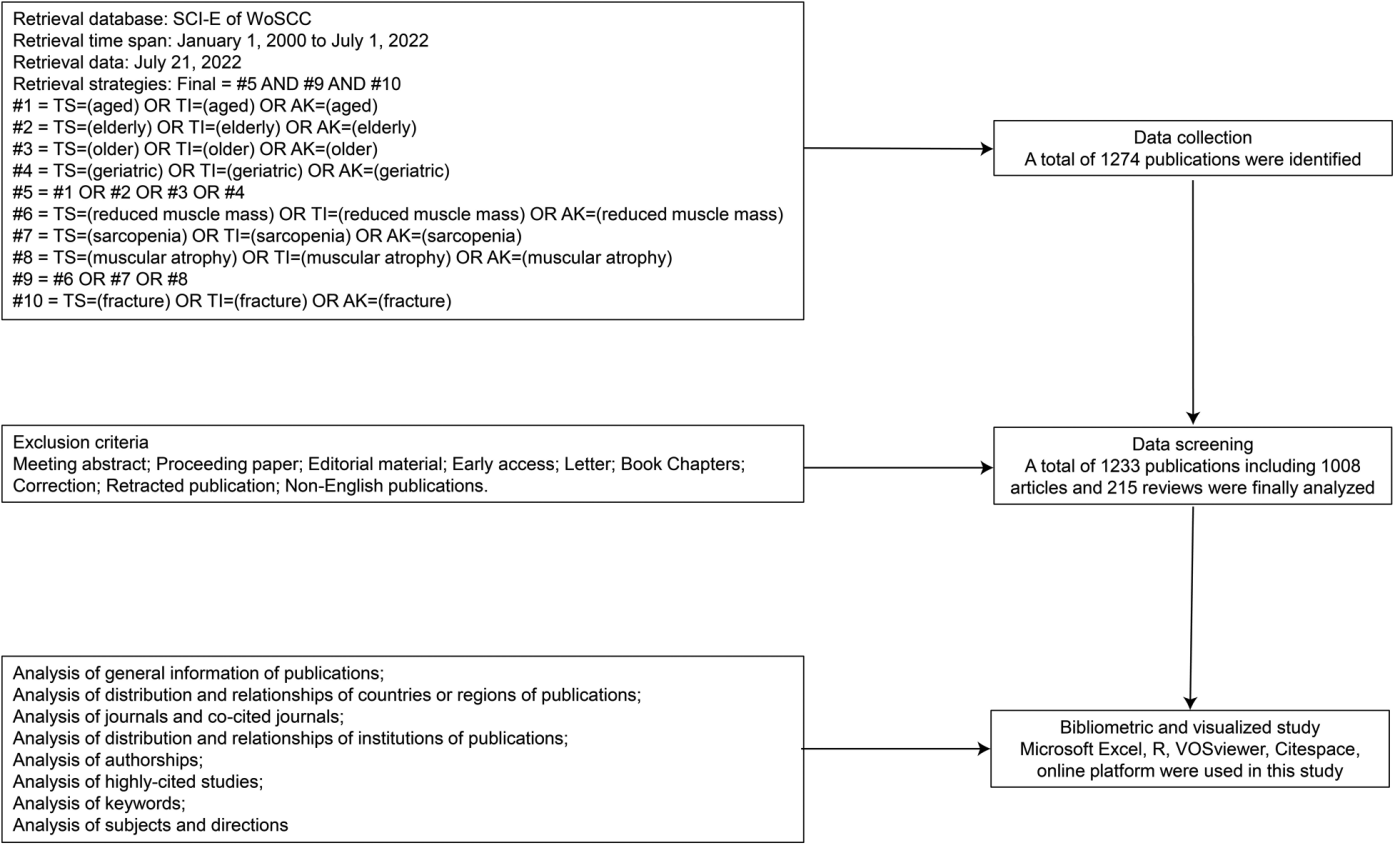
Flowchart of literature selection and analysis in this study.

The advanced search was used in this study, and the search strategies and formula were shown in Figure 1. The search terms for the elderly were: aged, elderly, older, and geriatric. The search terms for sarcopenia were: sarcopenia reduced muscle mass and muscular atrophy. The search term for hip fracture was hip fracture. The publications were identified by their topics, titles, and author keywords. The overall study included five phases as suggested in previous studies (21, 22): (a) identification of search range and keywords; (b) identification of time span and database; (c) refining of search criteria; (d) data transformation into visualization software; (e) data analysis.

### Screening strategies and data extraction

Inclusion criteria in this study were set as below: (a) articles focusing on sarcopenia in geriatric hip fracture; (b) documents types identified as article or review article. (c) documents with available relevant information. The exclusion criteria were based on the following: (a) meeting abstract; Proceeding paper; Editorial material; Early access; Letter; Book Chapters; Correction; Retracted publication; (b) non-English publications.

All information related to the identified publications was extracted from the WoS database: including title, authors, keywords, abstract, year of publication, nationality, affiliates, research direction, funding agencies, and H-index (23). The impact factor (IF) was collected based on the index provided by the 2021 Journal Citation Reports (JCRs). All the information of publication was screened and extracted by two authors independently, and any differences were discussed and resolved by a third author.

### Bibliometric and visualized analysis

Descriptive analysis was performed by using Microsoft Office Excel 2019 (Microsoft Corporation, Santa Rosa, CA, United States). All the characteristics of publications were summarized and ranked by using Microsoft Office Excel 2019 according to their publication year, nationality, affiliates, research direction, funding agencies, IF, the number of citations, and H-index. The predicted publication model was also established by the Trend Line function in Microsoft Office Excel 2019.

VOSviewer version 1.6.18 (Leiden University Center for Science and Technology Studies, Leiden, the Netherlands) was used to visualize the Network map of cited journals, co-cited journals, the collaboration of institute, co-cited authors, and keywords occurrence (24). There are three types of network maps provided by VOSviewer: the network map, the overlay map, and the density map. In the visualization map of VOSviewer, nodes represent the different parameters, such as authors, journals, and so on, and the size of nodes is related to the number of publications or references. The thickness of the line in the map represents the weighted total link strength (TLS), which means the degree of association between parameters.

CiteSpace (Chaomei Chen, Drexel University, United States) as a free visualizing software based on Java was used to analyze the collaboration between institutions and the cited relationship between authors (25). Moreover, the online bibliometric platform (https://bibliometric.com/) and the package Bibliometrix built in R were also used to analyze and visualize the collaboration relationship between countries and the citation relationship between journals.

## Results

### Analysis of general information of publications

**Figure 2:**
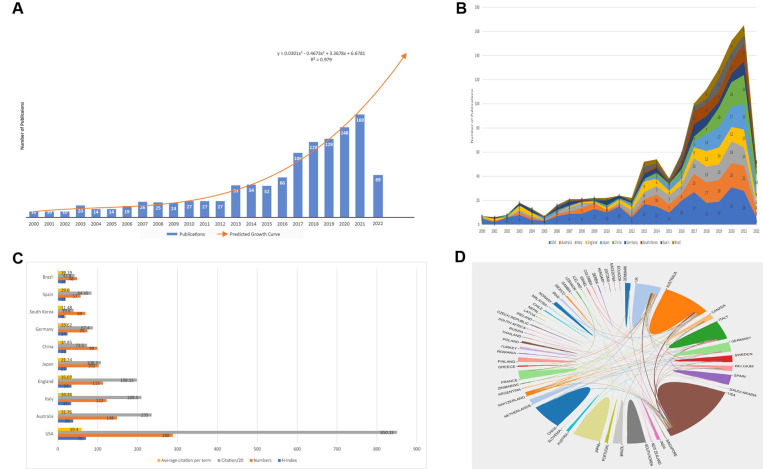
Analysis of general information and countries of publications. (**A**) The number of publications about sarcopenia and hip fractures in older patients from 2000 to 2022; (**B**) the number of publications of the top 10 countries with the largest number of studies from 200 to 2022; (**C**) the number of publications and citations, H-index, average citations per term of the top 10 most influential countries; (**D**) international cooperation among different countries or regions.

The 1,223 articles used in our study were written by 6,326 authors from 1,879 organizations in 60 countries, published in 388 journals, and cited 37,198 references from 5,422 journals. Between 2000 and 2021, the number of publications focusing on the relationship between sarcopenia and hip fractures in older patients had shown an overall upward trend. There were more than 100 studies published since 2017 (*n* = 106; 8.66%), and the number of publications reached the peak point in 2021 (*n* = 169; 13.82%) (Figure 2A). A predicted growth model equation was established by using Microsoft Office Excel 2019 to adjust the upward trend: *y* = 0.0301 × 3 − 0.4673 × 2 + 3.3678*x* + 6.6781, *R*^2^ = 0.979, in which *x* means the year and *y* means the predicted number of studies per year. Based on the predictive model we established, the numbers of publications are about 203 and 559 in 2022 and 2030, respectively. In the first half of 2022, 69 articles have been published.

### Analysis of distribution and relationships of countries or regions of publications

The trend of publications in the top 10 countries with the highest number of papers was shown in Figure 2B. The United States dominated the field of sarcopenia in geriatric hip fractures for the past 21 years, and since 2018 England, Japan and China experienced increasing publications. Figure 2C showed the characteristics of the 10 countries with the largest number of publications focusing on relationships between sarcopenia and geriatric hip fractures, including numbers of papers, H-index, Citations (transformed into citations/20), and average citations per term. The United States had the highest values of all the characters mentioned above and showed the strongest academic influence in this field. The international cooperation in different countries was shown in Figure 2D, and the line between countries means a cooperative relationship. United States collaborated most frequently with the UK, Australia, and Italy.

### Analysis of journals and co-cited journals

**Figure 3:**
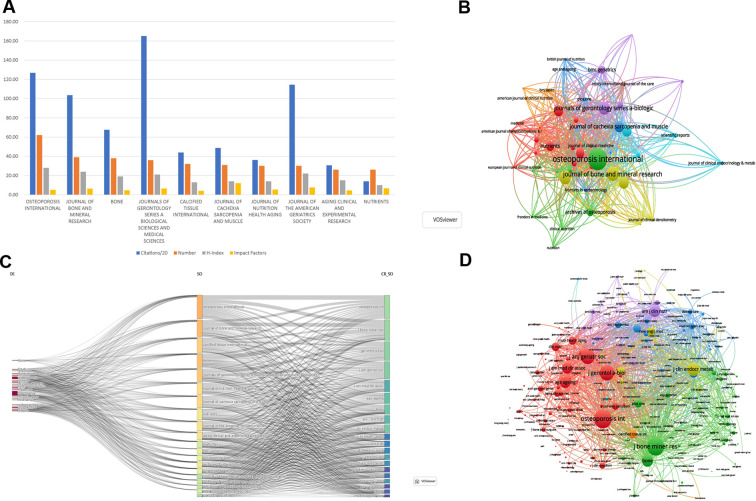
Analysis of journals and co-cited journals. (**A**) The general information of top 10 productive journals that published studies about sarcopenia and hip fractures in older patients; (**B**) network visualization map of journals that cited each other (generated by VOSviewer). The size of nodes is proportional to the cited times of journals. The thicker the lines between two nodes, the higher the frequency of their citation; (**C**) relationships between keywords, journals, and cited journals; (**D**) network visualization map of co-cited journals (generated by VOSviewer). The size of nodes is proportional to the co-cited times of journals. The thicker the lines between two nodes, the higher the frequency of their co-citation.

Of the 388 journals, 48 journals had at least 5 publications. The top 10 journals in terms of the number of studies about sarcopenia in elderly hip fractures were summarized in Figure 3A. The journal with the largest number of papers was Osteoporosis International (62 publications), which also had the highest H-index (H-index = 28). Journals of Gerontology Series A—Biological Sciences and Medical Sciences had been more cited than any other journals in this field (3,302 citations) (Figure 3A). Figures 3B,C showed the citation relationship between journals with publications in this field. Osteoporosis International had the largest total link strength (TLS) and Journals of Gerontology Series A—Biological Sciences and Medical Sciences had the largest citations. A total of 383 journals were cited at least 20 times. The co-citation relationship was shown in Figure 3D. The top 3 journals with the largest TLS were Journal of Bone and Mineral Research (TLS = 152,200), Osteoporosis International (TLS = 146,816), and Journal of the American Geriatrics Society (TLS = 106,800).

### Analysis of distribution and relationships of institutions of publications

**Figure 4:**
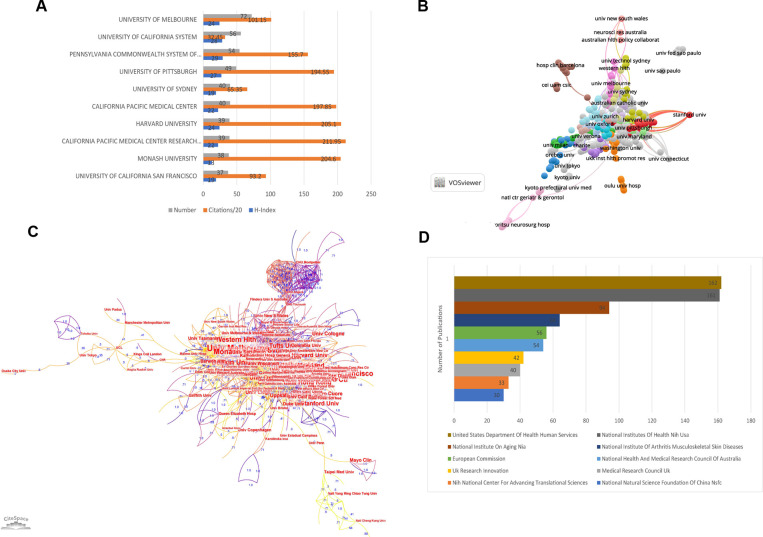
Analysis of institutions and funding organizations of publications. (**A**) The general information of top 10 productive institutions that published studies about sarcopenia and hip fractures in older patients; (**B**) Network visualization map of collaboration between institutions (generated by VOSviewer). The nodes present the institutions, and the color of the lines between two nodes presents the collaboration relationship between institutions; (**C**) network visualization map of collaboration between institutions (generated by Citespace); (**D**) the top 10 most active funding organizations for studies about sarcopenia and hip fractures in elderly.

The general information of the top 10 institutions with the largest number of publications was summarized in Figure 4A. The University of Melbourne published the biggest number of papers (number of papers = 72) focusing on sarcopenia in geriatric hip fractures while the California Pacific Medical Center Research Institute had the largest citations (number of citations = 4,239) (Figure 4A). The collaborative relationship between institutions was visualized in Figures 4B,C, and the top 3 institutions with the highest TLS were the University of Pittsburgh (TLS = 260), California Pacific Medical Center Research Institute (TLS = 194), and the University of California San Francisco (TLS = 183).

### Analysis of funding agencies

United States Department of Health Human Services funded the most publications (number of studies = 162), and the National Institute of Health funded a similar number of studies (number of studies = 161), followed by the National Institute on Aging (number of studies = 94). Half of the top ten funding agencies are located in the United States (Figure 4D).

### Analysis of authorships

**Figure 5:**
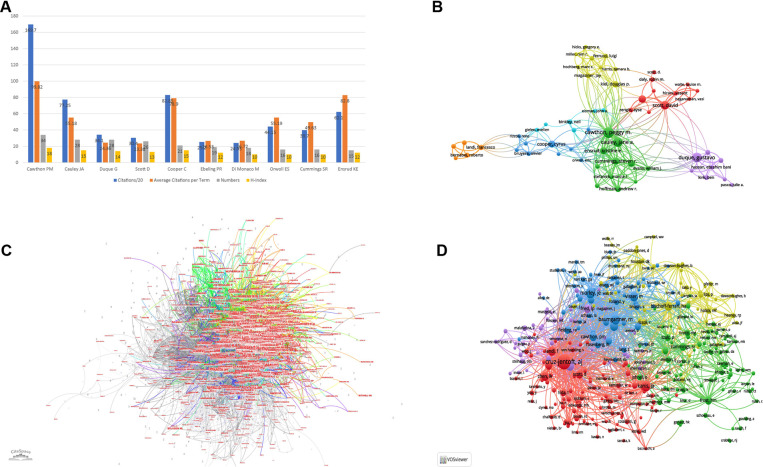
Analysis of authorships. (**A**) The general information of the top 10 productive authors with studies about sarcopenia and hip fractures in older patients; (**B**) network visualization map of collaboration between authors (generated by VOSviewer). The size of nodes is proportional to the collaboration frequency of authors, and the color of the lines between two nodes presents the collaboration relationship between authors. The thicker the lines between two nodes, the higher the frequency of their collaboration; (**C**) network visualization map of the cited relationship between authors (generated by Citespace); (**D**) network visualization map of co-cited authors (generated by VOSviewer). The size of nodes is proportional to the co-cited times of authors. The thicker the lines between two nodes, the higher the frequency of their co-citation.

The characters about citation, number of papers, and H-index of the most productive 10 authors focusing on sarcopenia and hip fractures in older adults were summarized in Figure 5A. Similarly, the top 10 authors with the largest citations were shown in Table 1. Cawthon PM was the most productive and influential author in the field, who published the most studies (number of studies = 34) in this field, and had the highest citations (citation = 3,394) and H-index (H-index = 18). Three authors have been cited more than 2,000 times: Cawthon PM (citation = 3,394), Kiel DP (citation = 2,276), and Harris TB (citation = 2,169). The collaborated relation between authors was visualized in Figure 5B. The partnership was divided into 7 clusters, and there were about 10 authors in each cluster. Every cluster was radiated by one author and few links were detected between different clusters. Figure 5C showed the citation relationship between authors, and there were a great number of influential authors who established the foundation for sarcopenia and elderly hip fractures. The co-cited author was visualized in Figure 5D: the top 3 authors with the highest TLS were Cruz-Jentoft AJ (TLS = 12,352), Morley JE (TLS = 6,630), and Janssen I (TLS = 6,349).

**Table 1:** The general information of the top 10 authors with the most citations.

	Citations	Average Citations per term	Numbers	H-index
Cawthon PM	3,394	99.82	34	18
Kiel DP	2,276	162.57	14	12
Harris TB	2,169	216.9	10	9
Ferrucci L	1,881	235.13	8	6
Cooper C	1,657	78.9	21	15
Alley DE	1,655	331	5	4
Guralnik JM	1,626	325.2	5	5
Cauley JA	1,545	55.18	28	15
Dam TTL	1,538	384.5	4	4
Ensrud KE	1,242	82.8	15	12

### Analysis of highly-cited studies

The top 10 most cited publications were summarized in Table 2. All these studies were published between 2000 and 2014 and acquired more than 300 citations. Half of them were published in journals focusing on geriatrics. An evidence-based study included 26,625 participants entitled “The FNIH sarcopenia project: rationale, study description, conference recommendations, and final estimates” published in Journals of Gerontology Series A—Biological Sciences and Medical Sciences had been cited 1,100 times and was the paper with the most cited numbers (26).

**Table 2:** The general information of the top 10 studies with the most citations.

Title	Journal	Author	Publication year	Citation
The FNIH Sarcopenia Project: Rationale, Study Description, Conference Recommendations, and Final Estimates	JOURNALS OF GERONTOLOGY SERIES A-BIOLOGICAL SCIENCES AND MEDICAL SCIENCES	Studenski, SA	2014	1,100
Lack of Exercise Is a Major Cause of Chronic Diseases	COMPREHENSIVE PHYSIOLOGY	Booth, FW	2012	1,088
Physical activity and bone health	MEDICINE / SCIENCE IN SPORTS / EXERCISE	Kohrt, WM	2004	601
Effects of testosterone on body composition, bone metabolism and serum lipid profile in middle-aged men: a meta-analysis	CLINICAL ENDOCRINOLOGY	Isidori, AM	2005	475
Frailty and risk of falls, fracture, and mortality in older women: The study of Osteoporotic fractures	JOURNALS OF GERONTOLOGY SERIES A-BIOLOGICAL SCIENCES AND MEDICAL SCIENCES	Ensrud, KE	2007	437
Health Outcomes of Sarcopenia: A Systematic Review and Meta-Analysis	PLOS ONE	Beaudart, C	2017	417
Epidemiology of sarcopenia	JOURNAL OF THE AMERICAN GERIATRICS SOCIETY	Melton, LJ	2000	387
Frailty in older men: Prevalence, progression, and relationship with mortality	JOURNAL OF THE AMERICAN GERIATRICS SOCIETY	Cawthon, PM	2007	320
Low-dose vitamin D prevents muscular atrophy and reduces falls and hip fractures in women after stroke: A randomized controlled trial	CEREBROVASCULAR DISEASES	Sato, Y	2005	311
Grip Strength Cutpoints for the Identification of Clinically Relevant Weakness	JOURNALS OF GERONTOLOGY SERIES A-BIOLOGICAL SCIENCES AND MEDICAL SCIENCES	Alley, DE	2014	303

### Analysis of keywords

**Figure 6:**
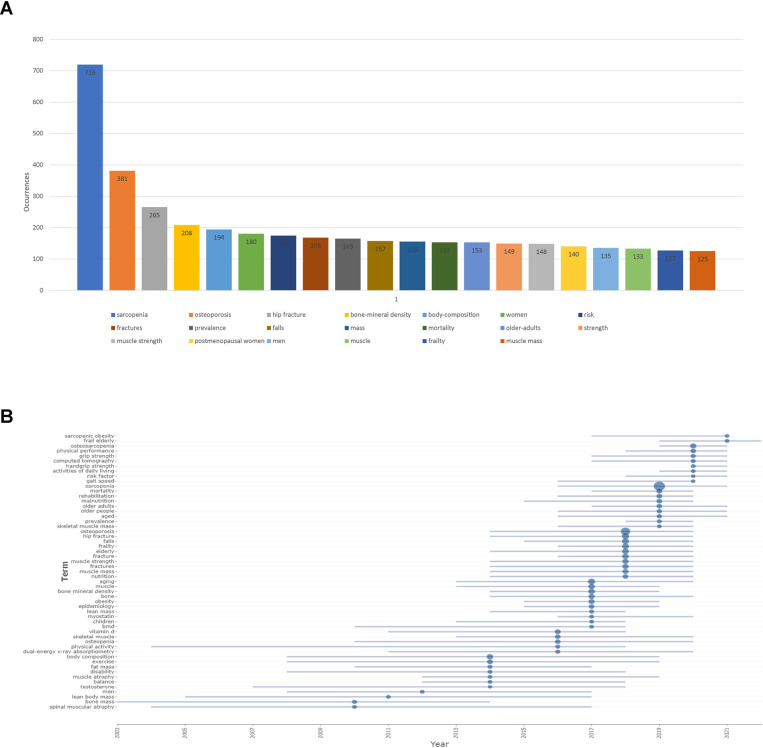
Analysis of keywords. (**A**) The general information of the top 20 common keywords in studies about sarcopenia and hip fractures in older patients; (**B**) trend of keywords from 2000 to 2022.

**Figure 7:**
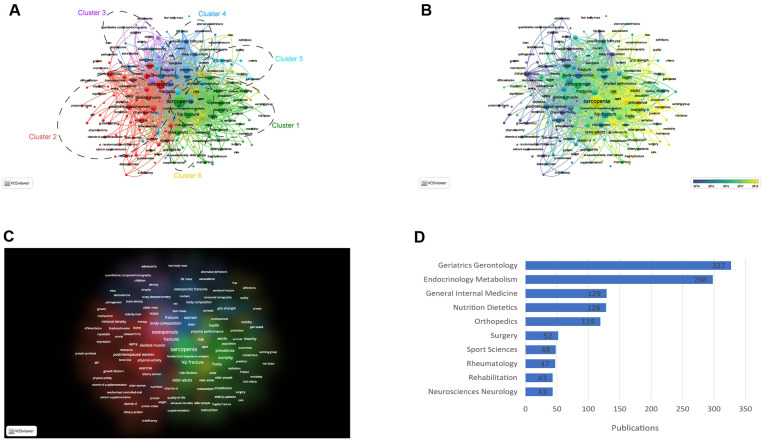
Analysis of keywords and analysis of subjects and directions. (**A**) Network visualization of the keywords co-occurrence analysis (generated by VOSviewer). The size of nodes is proportional to the co-occurrence times of keywords, and the color presents different clusters. The thicker the lines between two nodes, the higher the frequency of their co-occurrence; (**B**) average year map of keywords co-occurrence analysis (generated by VOSviewer). The size of nodes is proportional to the co-occurrence times of keywords, and the color presents on the average appearing year of each keyword. The thicker the lines between two nodes, the higher the frequency of their co-occurrence; (**C**) density distribution of keywords co-occurrence analysis (generated by VOSviewer). The density in the figure is proportional to the co-occurrence times of keywords; (**D**) distribution of subjects and directions of publications.

All keywords were extracted from 1,233 publications included in this study and analyzed by VOSviewer. The top 25 most common keywords generated by VOSviewer were summarized in Figure 6A. The keyword with the most frequency was ‘sarcopenia’ (719 times). Figure 6B showed the trend of the keyword in different years from 2000 to 2022, and the hotspots that researchers were focusing on in this field were changing. Firstly, spinal muscular atrophy and bone mass were studied, and for now, researchers are more interested in osteosarcopenia. As shown in Figure 7A, keywords were classified into 6 clusters: Cluster 1 (sarcopenia in aging), Cluster 2 (osteoporosis), Cluster 3 (bone density), Cluster 4 (body composition), Cluster 5 (physical performance), and Cluster 6 (risk factor). Keywords were categorized based on the year they appeared in Figure 7B. Before 2014, most studies focused on body composition while after 2018, publications about sarcopenia were more prominent. Similarly in Figure 7C, sarcopenia and osteoporosis were paid the greatest attention.

### Analysis of subjects and directions

Every publication in the WoSCC database was labeled with subjects and directions for searching conveniently in the database. The top 10 common subjects and directions of publications in this study were collected and summarized in Figure 7D. Consistent with the analysis of journals, Geriatrics Gerontology was the most prominent direction (number of publications = 327).

## Discussion

The SCIE database was searched to obtain the information from publications on sarcopenia and geriatric hip fractures, and many bibliometrics and visualization software including VOSviewer, Citespace, R software, and website (https://bibliometric.com) were used to analyze the data extracted from SCIE. Bibliometrics as an independent and newborn discipline could review the great number of literature quantitatively, and explore the potential hotspots that many researchers are interested in.

Hip fracture as a severe geriatric disease had addressed more and more attention in recent years due to its high morbidity and mortality (27). Geriatric hip fractures are always caused by low-energy injuries such as slip and fall, and ultimately, bone mass loss is the most essential cause (28). Many studies based on populations have suggested that patients with hip fractures treated with anti-osteoporosis medications may face an improved prognosis after surgery (29, 30). However, many risk factors were also reported to be associated with the risk and prognosis of geriatric hip fractures, such as supplementation of vitamin D3 (31), the neutrophil-to-lymphocyte ratio (32), ferritin (33), and so on. Sarcopenia as an age-related and complex disease had attracted much attention in recent years (34). However, the relationship between sarcopenia and hip fractures was still not fully understood, as well as sarcopenia and osteoporosis (14). Instead of comprehensive sarcopenia research, our study emphasizes sarcopenia in geriatric hip fractures. In this bibliometrics study, we clearly showed the trend and progress in the relationship between sarcopenia and hip fractures. The summary of the most influential authors, institutions, journals, publications, and popular keywords in this study may provide directions and suggestions for researchers who are interested in sarcopenia and hip fracture and may promote advanced understanding in this field.

For the global trends shown in Figure 1, the number of articles about sarcopenia in geriatric hip fracture has increased 16-fold from 2000 to 2022, which indicated that sarcopenia in hip fracture has gradually become a hotspot, and attracted more and more attention. As we all know, as the population in the world ages, geriatric diseases that may cause severe outcomes for patients have become a great challenge for society. It is foreseeable that the field of sarcopenia in hip fracture will continue to catch more researchers.

For the analysis of distribution and relationships of countries or regions of publications, the United States published the most documents in the world and had the most influence in this field. Until 2013, it was the United States that pioneered the studies about sarcopenia in geriatric hip fracture and published about half of the annual number of papers in this field. After 2014, sarcopenia in geriatric hip fracture gradually attracted more and more attention from authors over the world, especially in Japan, China, and Australia.

For the analysis of journals and co-cited journals, Osteoporosis International has published the most papers in this field. Osteoporosis International reports the frontiers of osteoporosis and other metabolic bone diseases. Journals of Gerontology Series A - Biological Sciences and Medical Sciences as the first journal on aging published in the United States had the most citations. Journal of Bone and Mineral Research had the highest TLS in the co-citation analysis. The analysis of journals and co-cited journals suggested that we could get the most advanced understanding of sarcopenia in geriatric hip fracture from the journals mentioned above.

For the analysis of distribution and relationships of institutions of publications, the institution with the most publications and the highest H-index was the University of Melbourne. However, California Pacific Medical Center Research Institute has the most citations. These two institutions may have a strong academic foundation in this field, and we could access the most leading-edge information from these institutions.

For the analysis of authorships, Cawthon PM was the most productive and influential author in the field. Cawthon PM managed many large, multi-center observational studies and oversaw the statistical Analyst Group at the Coordinating Center. She contributed a lot in the field of mobility, sarcopenia, frailty, osteoporosis, and age-related physical decline in older patients (35). A prospective cohort study that she was involved in indicated that the initial BMD screening may better predict the fracture in old community-dwelling men than repeated BMD screening (36). Studies based on population may report a plenty number of understandings of the relationship between sarcopenia and geriatric hip fractures, and rise more questions in the basic studies.

For the analysis of highly-cited studies, the most-cited paper about sarcopenia in the geriatric hip fracture is “The FNIH Sarcopenia Project: Rationale, Study Description, Conference Recommendations, and Final Estimates” published in Journals of Gerontology Series A - Biological Sciences and Medical Sciences in 2014, which had been cited 1,100 times (26). This study was a population study with large sample size and collected nine databases of community-dwelling older persons. The clinical cut-points for diagnosis of sarcopenia were identified in this study, and consistent with the above mentioned, the studies and attention to sarcopenia in geriatrics in this study were increasing rapidly since 2014. The top 2 most-cited papers in this field is a review article entitled “Lack of exercise is a major cause of chronic diseases” published in the Comprehensive Physiology (37). This paper reviewed the relation between exercise and chronic diseases in older patients, including sarcopenia, osteoporosis, and fragility fracture, and suggested that physical activity may prevent, or delay chronic diseases. Similarly, the third most cited publication also provided a summary of the efficiency of physical activity in bone health (38).

For the analysis of keywords, we could understand the development of keywords from 2000 to 2021. In the field of sarcopenia in geriatric hip fracture, the keywords were muscle atrophy, body composition, and exercise before 2014, and gradually, sarcopenia, osteoporosis, and hip fracture become the most popular keywords after 2014. Finally, all keywords were classified into six clusters. Cluster 1: sarcopenia in aging. Sarcopenia has been proved to be associated with many diseases in aging (39), and many studies are focusing on the theoretical links between aging and sarcopenia, and the potential efficiency of regular physical exercise in promoting life quality (40). Cluster 2: osteoporosis. Osteoporosis as the most common bone disorder in the world is related to an increased incidence of fragility fracture (41). In recent years, an increasing number of studies were focusing on the relationship between osteoporosis and sarcopenia (42), and many studies suggested that the mechanisms contributing to osteoporosis and sarcopenia may be the same (43). Cluster 3: bone density. Bone density is associated with osteoporosis and is the most prominent feature of osteoporosis. Patients with sarcopenia may face a high risk of decreased bone density, and a high incidence of hip fracture (44). Cluster 4: body composition. With a better understanding of sarcopenia, the relationship between body composition and geriatric hip fracture was attracting increasing attention in the world (45). Soft tissue body composition was found to be related to hip fracture and sarcopenia (46, 47). Cluster 5: physical performance. Sarcopenia, osteoporosis, and hip fracture are all musculoskeletal disorders, and physical performance is not only the essential method to diagnose musculoskeletal disorders, but also straightly reflects the therapeutic benefits (48). Cluster 6: risk factor. The risk factors of sarcopenia and hip fracture were well discovered in recent years. Many markers, including nutrition-related index and protein (49, 50), antioxidant enzymes (51), and inflammatory markers (52) may be associated with hip fracture.

There were some limitations to our study. Firstly, all the publications included in this study were collected from SCI-E, so many studies which were not indexed in SCI-E may be excluded. However, SCI-E is one of the most common databases in the world, and the literature indexed in SCI-E is of high quality. Secondly, non-English publications were excluded, which may cause some loss of studies. Moreover, for the analysis of citation frequency, some high-quality studies published in recent years may be cited less due to the short publication time. Lastly, sarcopenia as a newborn concept has undergone increasing development in the past 20 years, and some articles may have not been collected due to the unclear concept of sarcopenia in the early years.

## Conclusion

Our study reveals the global status of sarcopenia in geriatric hip fracture between 2000 and July 1, 2022. The United States was the most productive and influential country in this field. Osteoporosis International and Journals of Gerontology Series A—Biological Sciences and Medical Sciences were the journals with the largest number of publications and citations, respectively. The University of Melbourne, California Pacific Medical Center Research Institute, and the University of Pittsburgh were the leading institutions in this field. Consistent with the analysis of countries, funding agencies from the United States funded the most studies about sarcopenia in geriatric hip fracture. Cawthon PM was the most influential and productive author, and Geriatrics Gerontology was the most prominent direction in this field. Keywords were classified into 6 clusters: Cluster 1 (sarcopenia in aging), Cluster 2 (osteoporosis), Cluster 3 (bone density), Cluster 4 (body composition), Cluster 5 (physical performance), and Cluster 6 (risk factor). All in all, our study provided valuable information for researchers to understand the current hotspots and research trends on sarcopenia in geriatric hip fracture.

## Data Availability

The raw data supporting the conclusions of this article will be made available by the authors, without undue reservation.
